# The Effect of Computer Tablets on the Need for Medical Anxiolysis in Children in an Ambulatory Surgical Center

**DOI:** 10.7759/cureus.42553

**Published:** 2023-07-27

**Authors:** Kerry H Farlie, Thomas M Austin, Sandra N Gonzalez, Christopher M Edwards, Nikolaus Gravenstein, Fred C Dooley

**Affiliations:** 1 Anesthesiology, University of Florida College of Medicine, Gainesville, USA

**Keywords:** audiovisual distraction, tablet, midazolam, ambulatory surgery, anxiolytics, pediatric anesthesiology, perioperative anxiety

## Abstract

Background

Preoperative anxiety is common in children undergoing surgery. When anxiety is identified or suspected, there are several strategies typically used to manage it. Perhaps the most common is anxiolytic premedication or parental presence at induction. Medications such as midazolam have been associated with adverse effects, such as a slower wakeup, and require timing of administration, while parental presence can be disturbing to the parent and divert the attention of the operating room team. A more recent option is distraction via electronic tablets. The purpose of this study was to retrospectively investigate and quantify any change in the use of midazolam, the most common anxiolytic approach at our institution, and any change in the length of time in the post-anesthesia care unit (PACU) following the introduction of tablet computers to a pediatric ambulatory surgical center.

Methods

We conducted an IRB-approved retrospective chart review of 13,790 pediatric patients ages one to 18 undergoing outpatient elective surgeries at the University of Florida (UF) Children’s Surgical Center over a five-year period. A univariate analysis was conducted using the Fisher’s Exact test and interrupted time series analysis to determine differences between midazolam administration and PACU times, with interruption occurring at tablet implementation. A multivariable analysis and sensitivity analyses were performed to confirm the findings of the univariate analysis.

Results

On univariate analysis, tablet availability was associated with both a decreased preoperative oral midazolam administration (odds ratio (OR) 0.158, 95% confidence interval (CI): 0.140 to 0.179, P-value <0.001) and a decreased PACU length of stay (-17.4 min, 95% CI: -19.6 to -15.3 min, P-value <0.001). The association with decreased preoperative midazolam administration held after multivariable analysis (adjusted OR 0.207, 95% CI: 0.154 to 0.278, P-value <0.001), but PACU length of stay was not statistically significant (-9.1 min, 95% CI: -20.6 to 2.4, P-value = 0.12). These results were confirmed on sensitivity analysis, with tablet availability continuing to be associated with decreased preoperative oral midazolam administration but not with reduced PACU length of stay.

Conclusion

Our results demonstrate that computer tablets were associated with a significant decrease in the frequency of midazolam administration and consequently may reduce preoperative pediatric anxiety. We did not find an associated change in PACU length of stay following the introduction of tablets. Tablets present a unique distraction alternative to chemical anxiolysis for institutions seeking to reduce medication use in pediatric patients.

## Introduction

Preoperative anxiety is present in the majority of pediatric patients undergoing surgery with general anesthesia [1]. Fear of pain, separation from a parent, an unfamiliar environment, and loss of control can all lead to anxiety in children prior to sedation [2]. While reducing pediatric preoperative anxiety has clear intrinsic value, it may also help to improve recovery, as literature has demonstrated an association between higher preoperative anxiety and a greater frequency of pain, sleep disturbances, and delirium following surgery [3].

Conventionally, anxiolytics are used to prevent and treat childhood preoperative anxiety [4]. Oral midazolam is the pharmacological gold standard for treating preoperative anxiety, representing 85% of anxiolytics given to children going into the operating room [5,6]. However, midazolam can result in several adverse effects, including decreased oxygen saturation, respiratory rate, and blood pressure [7]. Premedicating with midazolam has also been shown to result in increased perioperative respiratory adverse events, especially airway obstruction [8]. Additionally, some sources suggest that midazolam may prolong recovery time [6]. Given these concerns, it is attractive to explore alternatives to pharmacologic anxiolytics for the treatment of preoperative anxiety in children.

Distraction is a well-established technique for reducing anxiety prior to surgery [4,9]. Specifically, there is a growing body of literature highlighting the role that technology can play in distracting children and successfully alleviating preoperative anxiety. Studies have shown that handheld video games, DVD players, cartoons, music, virtual reality, and even video glasses significantly reduce levels of anxiety in children preparing to undergo surgery [6,10-12]. In particular, when tablet-based distraction was compared to midazolam, improvements were seen in emergence delirium and length of stay, in addition to preoperative anxiety [13].

The University of Florida (UF) opened a new pediatric ambulatory surgical center in April 2016. Tablet computers loaded with popular movies and video games were purchased and made available to patients. Over the coming months, the anesthesiology faculty and staff at the surgical center noticed that pediatric patients were less frequently requiring premedication. This retrospective study investigated the frequency of midazolam use and length of time in the PACU among pediatric patients presenting for procedures at our outpatient surgical center.

## Materials and methods

Tablet implementation in an outpatient surgical setting 

Tablet computers were made available for all patients to use while preparing for surgery at the UF Children's Surgical Center (CSC) when the new facility opened on April 1, 2016. These tablets were maintained by the information technology (IT) department, set up with a common password protection system, and loaded with games and movies that are popular among various age groups. In the interest of safety and accountability, the tablets were not able to browse the internet. The preoperative nursing staff offered tablets to patients with parental permission at the start of the patient/parent interview process, and they could be used throughout their stay, including in the recovery room. The tablet was signed out to each patient and passed along as part of the standard nursing handoff. The tablet computers were locked in protective cases that were sanitized after each patient's use. Tablets were stored overnight in a locked charging station for safekeeping.

Data collection

An IRB-approved retrospective observational chart review of 13,790 patients was conducted. All pediatric patients one to 18 years old who had an outpatient elective surgery under general anesthesia at the University of Florida, Children’s Surgical Center (CSC) between October 1, 2015, and September 30, 2020, were included in the study. Patients were divided into two cohorts: pre- and post-introduction of tablets. Data extracted from the EPIC electronic medical record included patient age, presence or absence of midazolam administration in the preoperative phase of care, surgical service, procedure type, post-anesthesia care unit length of stay, total length of stay, and length of surgery. Surgical procedures primarily included circumcisions, endoscopies, tonsillectomies, and tympanostamies.

Statistical analysis

The Fisher’s Exact test or the independent t-test was used to determine univariate proportional differences or mean differences between the two cohorts, respectively. Since this was a before-after study design, interrupted time series analyses were utilized to determine differences between midazolam administration and PACU times, with the interruption occurring at the tablet implementation (April 1, 2016) [14]. To account for possible unmeasured correlations between observations from different time points, multivariable generalized estimation equation (GEE) models were employed with autoregressive 1 (AR(1)) covariance matrices. Variables included in both models were tablet implementation (yes/no), pre-intervention time (month), and post-intervention time (month). Sensitivity analyses were performed by creating similar GEE models for patients aged eleven or less. All two-sided p-values less than 0.05 were considered statistically significant. R statistical software (version 4.2.3) was used in this analysis.

## Results

**Figure 1:**
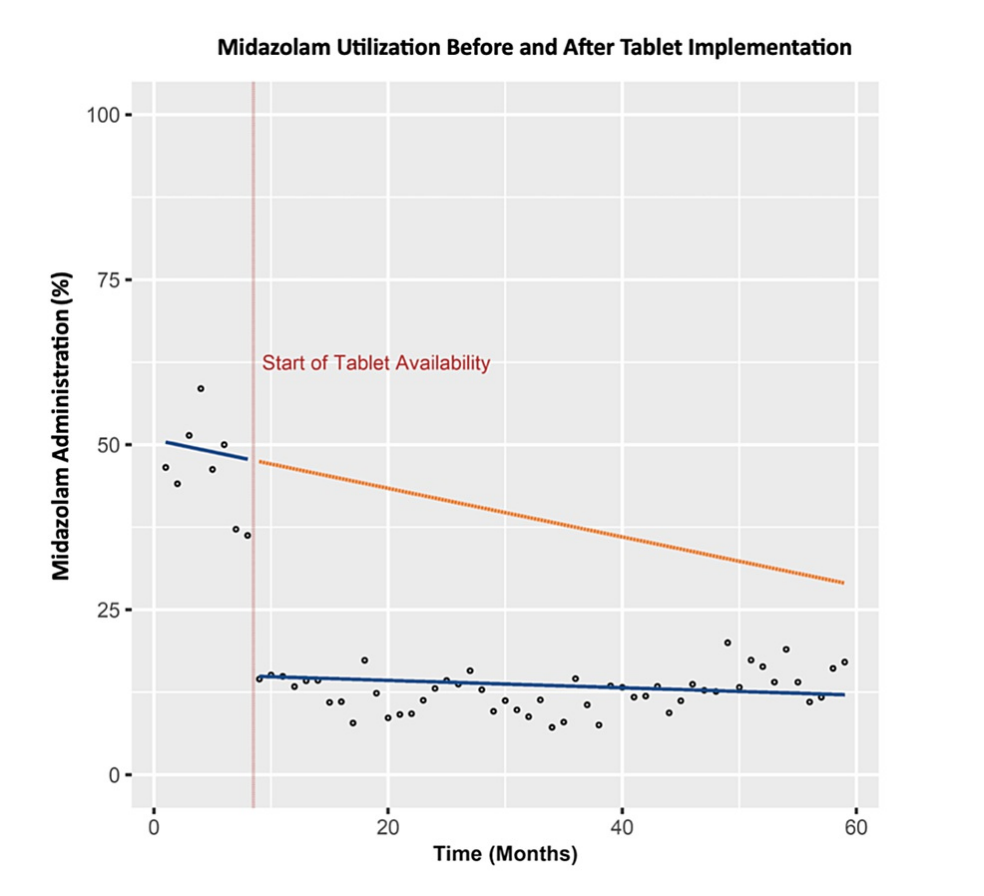
Comparison of midazolam administration before and after the introduction of computer tablets Scatter plot of the rate of oral midazolam administration over time. The vertical red dashed line depicts the start of tablet availability at the ambulatory center. Solid blue lines represent linear regression lines before and after tablets became available, while the dashed orange line signifies the hypothetical trend in oral midazolam administration rate if tablets were never introduced.

In this analysis, 13,790 pediatric patients were included, with 1,227 patients and 12,563 patients in the pre-tablet and post-tablet cohorts, respectively (Table 1). The average ages of the patients in both cohorts were 6.0 ± 4.8 years, with a strong majority being less than 11 years old (Table 1). On univariate analysis, tablet availability was associated with both decreased preoperative oral midazolam administration (odds ratio (OR) 0.158, 95% CI: 0.140 to 0.179, P-value <0.001, Table 2 & Figure 1) and decreased PACU length of stay (-17.4 min, 95% CI: -19.6 to -15.3 min, P-value <0.001, Table 2). Although the association with decreased preoperative midazolam administration held after multivariable analysis (adjusted OR 0.207, 95% CI: 0.154 to 0.278, P-value <0.001, Table 2), PACU length of stay was not statistically significant (-9.1 min, 95% CI: -20.6 to 2.4, P-value = 0.12, Table 2). These results were confirmed on sensitivity analysis, with tablet availability continuing to be associated with decreased preoperative oral midazolam administration (adjusted OR 0.160, 95% CI: 0.114 to 0.223, P-value <0.001, Table 2) but not associated with PACU length of stay (-2.7 min, 95% CI: -8.6 to 3.2 min, P-value = 0.37, Table 2). 

**Table 1:** Demographics and perioperative variables* Preop: preoperative; PACU: post-anesthesia care unit *Nominal variables are expressed as count (percentage), while continuous variables are expressed as mean ± standard deviation. Univariate analyses were conducted utilizing the Wilcoxon rank-sum test or Fisher Exact test, as appropriate for the distribution.

	No tablets available (n = 1227)	Tablets available (n = 12563)	P-value
Age (years)	6.0 ± 4.8	6.0 ± 4.8	0.70
Age ≤ 11 years (%)	1012 (82.5%)	10444 (83.1%)	0.55
Preop midazolam (%)	610 (49.7%)	1698 (13.5%)	<0.001
Surgical duration (minutes)	68.5 ± 52.1	68.6 ± 51.6	0.95
PACU length of stay (minutes)	94.7 ± 54.1	77.3 ± 34.3	<0.001

**Table 2:** Association between tablet availability and outcomes OR: odds ratio; Coef: coefficient; CI: confidence interval; PACU: post-anesthesia care unit ^§^Based on simple logistic and linear regression for midazolam and PACU length of stay, respectively. P-values <0.05 are considered statistically significant. ^¥^Based on interrupted time series analysis with the use of multivariable generalized estimation equation (GEE) models with autoregressive 1 (AR(1)) covariance matrices. Variables included in both models were tablet implementation (yes/no), pre-intervention time (month), and post-intervention time (month). P-values <0.05 are considered statistically significant.

			^§^Unadjusted			^¥^Adjusted		
Outcomes	Levels		OR / Coef	95% CI	P-value	OR / Coef	95% CI	P-value
Midazolam administration (%)		Tablet availability vs none (referent)	0.158	0.140 to 0.179	<0.001	0.207	0.154 to 0.278	<0.001
PACU length of stay (min)		Tablet availability vs none (referent)	-17.4	-19.6 to -15.3	<0.001	-9.1	-20.6 to 2.4	0.12

## Discussion

We observed a dramatic decrease in midazolam administration at our pediatric ambulatory surgical center following the introduction of electronic tablets. This reduced rate of midazolam administration persisted for four years following the introduction of tablets. Our findings demonstrate that electronic distraction may be an effective means of decreasing premedication with midazolam. Though we did not find a significant change in PACU length of stay, decreased use of pharmacologic anxiolytics may improve long-term patient recovery following general anesthesia. This study at a large tertiary care ambulatory surgical center supports many previous studies that have demonstrated that tablets and other modes of electronic distraction significantly decrease the frequency of midazolam given for preoperative anxiety in children [12,15,16]. The reduced percentage of children requiring pretreatment with midazolam after the introduction of tablets suggests that the tablets were successful in reducing preoperative anxiety in this cohort, which is consistent with the current literature [6,10-12].

We recognize that there are several limitations to this study. First, we did not assess anxiety levels in patients in a formal way. We assessed in our usual manner: children who appeared anxious received midazolam, and children who did not appear anxious did not. We have no data about behavior at induction, but studies comparing electronic tablets to various different forms of distraction and behavior at induction abound in the literature. We have no data regarding the incidence of postoperative delirium or adverse effects of midazolam in these patients, but PACU length of stay was not negatively impacted in length, as one might come to expect if delirium or other adverse reactions were to occur. We also looked at all children under the age of 18 in this study. Although different ages of children will have varying levels of anxiety and be at different levels of cognitive development, the age groups were equally represented in those who had surgery prior to tablet availability and those after tablet availability. It is our local practice to start IVs starting at age 12 preoperatively and for those under 12 years of age to receive an inhalational induction, so we further subdivided our original age limit to those under the age of 12. Both the groups under the age of 12 and under 18 were equally represented in the groups before and after tablet implementation.

We have no child life specialists, and parental presence at induction is not a usual occurrence at our facility. We also have no measures of patient adaptability to using tablets or taking midazolam to compare. Our patient population was also limited because we only looked at patients who were undergoing same-day elective surgeries at a free-standing outpatient surgical center. Despite these limitations, we still believe that our study has merit in its original state. Since the study occurred over a five-year period, there may be the possibility of differences in variables between cohorts not accounted for in our analysis that could confound our results. However, the interrupted time series model that was employed should minimize this potential bias. Rather than focusing on all individual factors that may be associated with changes in midazolam use, the statistical model analyzed overall rates of midazolam use over time, specifically in relation to the introduction of computer tablets. Any change in factors associated with midazolam administration over the course of the study would be controlled for in this analysis.

Our study demonstrates a reduction in midazolam use after the computer tablets became available, which is something that we had suspected anecdotally and that prompted the data collection approximately one year after the tablets became available. We continued gathering data retrospectively to see if any further changes occurred. If midazolam use continued to decline, one might suspect providers were under-recognizing and under-treating anxiety, and PACU times might consequently increase due to an increased incidence of delirium. If midazolam use increased again over time, then other conclusions might be drawn. Although causation cannot be established, midazolam use did decrease. Induction behavior was not noted to deteriorate, and we did not observe an increase in PACU length of stay. Our report represents an organic description of the impact of the implementation of tablet computers on our practice. We believe that our findings confirm what almost any parent would tell you: children are distracted by tablet computers. In our setting, this distraction resulted in less midazolam use and is of interest and applicable to many other institutions with similar resources that are examining alternatives to pharmacologic anxiolysis.

The fear with any attempt to decrease pharmacologic agents for anxiety is under-diagnosing and under-treating anxiety. We recognize that without a validated tool to recognize and treat anxiety in children, the decision to medicate a child is not a perfect science. As such, our facility is in the process of implementing a tool to standardize this process, which is especially important in an academic setting so that learners with different backgrounds can be placed on a more level playing field in recognizing and addressing preoperative anxiety. Future goals include predicting which patients are most likely to experience preoperative anxiety and determining the most appropriate treatment algorithm based on individual patient characteristics.

The electronic tablets have proven a good investment for our pediatric ambulatory surgical center. They are still being used at the CSC, and we have had an adequate supply of electronic tablets to give to each pediatric patient scheduled for surgery. We have not had to buy any additional electronic tablets or replace any due to damage in the seven years since they were originally purchased. Some tablets have been found in linens in our laundry department, but these have almost all been recovered without damage, and only one has been lost over the seven years since their introduction. The tablets have required minimal upkeep. Aside from the original purchase, no additional money has been spent on the tablets.

## Conclusions

In summary, our results demonstrate that the introduction of computer tablets to our pediatric ambulatory surgical center was associated with a significant decrease in the frequency of premedication with midazolam for preoperative anxiety in children. This observed decrease in midazolam administration persisted for four years after tablets were made available to all pediatric patients. Tablet availability and decreased midazolam usage have not significantly impacted the PACU length of stay. We believe this cohort is the largest observational study to date to evaluate the relationship between electronic distraction and anxiolytic administration, though further studies are needed to adequately quantify the resulting change in anxiety. Pediatric ambulatory surgical centers should consider electronic tablets as a cost-effective means of reducing reliance on pharmacologic anxiolytics.
